# The Methanol Extract of *Polygonatum odoratum* Ameliorates Colitis by Improving Intestinal Short-Chain Fatty Acids and Gas Production to Regulate Microbiota Dysbiosis in Mice

**DOI:** 10.3389/fnut.2022.899421

**Published:** 2022-05-12

**Authors:** Xuewei Ye, Xionge Pi, Wenxin Zheng, Yingxin Cen, Jiahui Ni, Langyu Xu, Kefei Wu, Wei Liu, Lanjuan Li

**Affiliations:** ^1^State Key Laboratory for Diagnosis and Treatment of Infectious Diseases, Collaborative Innovation Center for Diagnosis and Treatment of Infectious Diseases, Zhejiang University, Hangzhou, China; ^2^Key Laboratory of Pollution Exposure and Health Intervention of Zhejiang Province, Department of Basic Medical Sciences, Shulan International Medical College, Zhejiang Shuren University, Hangzhou, China; ^3^Institute of Plant Protection and Microbiology, Zhejiang Academy of Agricultural Sciences, Hangzhou, China

**Keywords:** *Polygonatum odoratum*, DSS-induced colitis mice, gas production, SCFAs, microbiota

## Abstract

The potential impacts of methanol extract from *Polygonatum odoratum* on (YZM) colonic histopathology, gut gas production, short-chain fatty acids (SCFAs), and intestinal microbiota composition were evaluated with dextran sulfate sodium (DSS)-induced colitis mice in this study. These results indicated that YZM increased colon length and ameliorated colonic histopathology in DSS-induced colitis mice. Moreover, YZM administration reversed intestinal microbiota compositions leading to the inhibition of H_2_S-related bacteria (e.g., *Desulfovibrionaceae*) and the lower level of H_2_S and higher contents of SCFA-related bacteria (e.g., *Muribaculaceae*). Taken together, the effects of methanol extract from *Polygonatum odoratum* are studied to provide new enlightenment and clues for its application as a functional food and clinical drug. Our study first revealed the relationship between intestinal gas production and key bacteria in ulcerative colitis.

## Introduction

Inflammatory bowel disease (IBD) is a relapsing non-specificity inflammatory condition that results from a chronic disorder of the gastrointestinal mucosa immunity system, including Ulcerative colitis (UC) and Crohn’s disease (CD) (1). Recent investigations indicate that the prevalence rate of IBD has also increased sharply, especially among younger people. Typical clinical manifestations of IBD include abdominalgia, enterorrhagia, hematochezia weight loss, and recurrence due to inflammatory cell infiltration. Some studies have found that the causes of UC include pathogenic microorganism infection, genetic susceptibility, intestinal microbiome imbalance, and intestinal mucosal barrier defect. However, at present, the pathological mechanism is not clear. The current clinical treatments (sulfasalazine and mesalazine) are mainly to relieve the symptoms of the disease but accompany adverse impacts (2). Thus, developing a safe and high efficacy treatment to remiss IBD is urgently required.

The intestinal mucosal barrier prevents disease-causing substances and bacteria from entering the bloodstream. The microbial barrier composed of a large number of microbial colonies is crucial in the development of IBD. Extensive research has suggested that changes in colon microflora composition caused by an abnormal increase in pathogenetic bacteria or deficiency of probiotics are associated with IBD. Accompanied by intestinal microbiota disturbance, the alteration of metabolites such as short-chain fatty acids (SCFAs) and gas affect colitis’s progression, which is intimately related to colon cancer (3). Although the exact mechanisms related to microorganisms remain to be elucidated, emerging evidence suggests that various natural substances contribute significantly to the improvement of IBD by regulating gut microbiota (4).

Dietary intake is closely associated with the pathogenesis and prevention of IBD without adverse influence (5). The interaction between dietary nutrients and intestinal immunity is very complex, referring to the immune response and the regulation of intestinal microflora composition (6). Ingested natural nutrients can accumulate and interact with intestinal microbiota (7, 8). As the sole input resource for intestinal microbiota, dietary intake has a significant impact on the intestinal microbiome composition (9, 10). Therefore, natural nutrients, which are characterized by low toxicity, multiple components, and targets, play an important role in potentially maintaining microbial homeostasis in patients with IBD with long-term use.

*Polygonatum odoratum* (Mill.) Druce (YZ) is a perennial herbaceous plant in the Liliaceae family that is widespread in East Asia and Europe. The root of YZ is a sweet and light food, and it is also traditional Chinese medicine that can relieve intestinal problems (11). YZ contains a variety of active substances, namely, flavonoids, terpenoids, phenols, coumarin alkaloids, and organic acids (12). Flavonoids, as an antioxidant, can delay aging, inhibit viral activity, inhibit bacterial reproduction, prevent cancer, and enhance the immune system. In addition, flavonoids are considered a functional factor in healthy foods. Resveratrol, a polyphenol, has a protective effect on acute or chronic colitis in different models, downregulating inflammatory biomarkers and reducing clinical symptoms (13).

Methanol extract of *Polygonatum odoratum* (YZM) was prepared to elucidate its protective effect on UC and further explore its related mechanism in our research. We assessed the impacts of YZM on body weight, colon length, colon lesion degree, intestinal microbiota composition, and metabolites of DSS-induced colitis mice. Our investigation provides insights into the influence of YZM on colitis related to the interactions of microbiota and metabolites. This evidence could support the new therapeutic and preventive avenues for IBD.

## Materials and Methods

### Preparation of the YZM

*Polygonatum odoratum* was derived from Bozhou Zhongyitang Traditional Chinese Medicine Sales Co., Ltd. The crushed medicinal materials were extracted with methanol at room temperature and repeated three times. The extracts were concentrated and named YZM (yield 20%), the methanol extract from *Polygonatum odoratum*. YZM was dried under vacuum conditions (decompress distillation and vacuum desiccation) for biological tests and stored at 4°C.

### Compositions Analysis of YZM

Orbitrap MS (Thermo Fisher Scientific, United States) with the ACQUITY UPLC^®^ HSS T3 (150 mm × 2.1 mm, 1.8 μm, Waters) column was applied for UHPLC-MS analyses. The mobile phase flow rate was 0.25 ml/min; solvent A was composed of 0.1% formic acid in the water, and solvent B was composed of 0.1% formic acid in acetonitrile. The elution condition was as follows: 2% B for 1 min, 2–50% B for 8 min, 50–98% B for 4 min, 98% B for 1.5 min, 98–2% B for 1.5 min, and 2% B for 6 min. The electrospray ionization mass spectrometry (ESI-MS) setting was as follows: positive mode (3.5 kV), negative mode (−2.5 kV), auxiliary gas at 30 units, capillary temperature at 325°C (14, 15). The mass spectra were scanned at a resolution of 60,000 from 100 to 1,000 m/z.

### Design of Experiments

Male BABL/c mice (6–8 week old, specific pathogen-free grade, 20 ± 2 g, *n* = 30) were purchased from the Zhejiang Academy of Medical Science and raised at the Zhejiang Academy of Agricultural Sciences. Mice were subjected to an artificial LED light cycle (12-h light/12-h dark) and ambient temperature (24–26°C) indoors. Mice were free to intake standard food and water for 2 weeks under laboratory conditions. The mice were randomly divided into five groups: a control group treated with phosphate-buffered saline (PBS), a model group treated with PBS, a positive control group treated with salicylazosulfapyridine (SASP, 50 mg/kg), a YZM-L group treated with a low concentration of YZM (YZM-L, 200 mg/kg), and a YZM-H group treated with a high concentration of YZM (YZM-H, 400 mg/kg). All drugs were administered by intragastric administration. The control group supplied sterile water. The other groups were supplied with 3% DSS solution for 8 days to induce colitis. All mice were sacrificed on day 9.

### Evaluation of Disease Activity Index Score

The disease activity index (DAI) score is a common index to assess the colitis severity of mice. All mice were evaluated for weight loss, stool character, and fecal occult blood. The average of these scores was assigned according to DAI criteria (Table 1) (5).

**TABLE 1:** Criteria for DAI.

DAI score	Weight loss	Dropping consistency	Occult/blood bleeding
0	None	Normal	Normal
1	1–5		
2	5–10	Loose droppings	Occult blood positive
3	10–20		
4	>20	Diarrhea	Blood bleeding

### Evaluation of Colonic Pathological Changes

Fresh colon tissues were fixed in 4% buffered paraformaldehyde for 48 h and then embedded in paraffin. After stained with hematoxylin–eosin (H&E), photomicrographs were obtained using a microscope for histological examination and histopathological score (Table 2) on 4-μm-thick sections (16).

**TABLE 2:** Histological scoring system.

Histological score	Degree of inflammation	Infiltration of inflammatory cells	Degree of damage to the crypt	Crypt abscesses	Degree of submucosal edema	Reduction of goblet cells	Degree of epithelial hyperplasia
0	Normal	Normal	Normal	Normal	Normal	Normal	Normal
1	Mucosa	Unifocal	Basal 1/3 of crypt	Unifocal	Unifocal	Unifocal	Unifocal
2	Submucosa	Multifocal	Basal 2/3 of crypt	Multifocal	Multifocal	Multifocal	Multifocal
3	Muscular	Suffuse	Entire crypt		Suffuse	Suffuse	Suffuse
4	Serous		Damage to the crypt and ulceration				

### Fermentation With Mice Fecal

Fresh fecal samples (0.24 g) were homogenized with 2.4 ml of 0.1 M PBS (pH 7.0). After filtration by 300 mesh filter sieves, the supernatants were transferred into modified medium (17). The recipe for 1 L was as follows: starch, 8 g; yeast extract 4.5 g; tryptone, 6 g; L-cysteine hydrochloride, 0.8 g; bile salt, 0.4 g; hemin, 0.05 g; NaCl, 4.5 g; MgCl_2_ ⋅6H_2_O, 0.45 g; CaCl_2_ ⋅6H_2_O,0.2 g; KCl, 2.5 g; KH_2_PO_4_, 0.4 g; 1 ml of Tween-80 and 2 ml of a solution of trace elements (g/L, MgSO_4_ ⋅7H_2_O, 3.0; MnCl_2_ ⋅4H_2_O, 0.32; FeSO_4_ ⋅7H_2_O, 0.1; CoSO_4_ ⋅7H_2_O, 0.18; CaCl_2_ ⋅2H_2_O, 0.1; ZnSO_4_ ⋅7H_2_O, 0.18; CuSO_4_ ⋅5H_2_O, 0.01; and NiCl_2_ ⋅6H_2_O, 0.092).

### Gas Production

The gas detector measured gas composition after fermentation. Carbon dioxide (CO_2_), hydrogen (H_2_), methane (CH_4_), hydrogen sulfide (H_2_S), and ammonia (NH_3_) were measured simultaneously after being incubated at 37°C for 24 h (17).

### Analysis of Short-Chain Fatty Acids

Gas chromatography (Shimadzu, Japan) with DB-FFAP column (0.32 mm × 30 m × 0.5 μm, Agilent Technologies, United States) was used to quantify the SCFAs. The operation condition was as follows: the flow rate of nitrogen carrier gas: 19.0 ml/min; split ratio: 1:10, the temperature of both detector and injection port: 250°C. Crotonic acid was used as an internal standard (17).

### The Intestinal Microbiota Analysis

After the fecal sample’s DNA was extracted, and the V3-V4 fragment of the bacterial 16S rDNA gene was amplified with primers (341F/805R). Agilent 2100 Bioanalyzer (Agilent, United States) evaluated the amplicon library. Then, sequencing was performed on the NovaSeq PE250 platform. According to the specific barcode of the sample, the paired-end data were overlapped using the FLASH (v1.2.11). The feature table and sequence, obtained with DADA2 (Divisive Amplicon Denoising Algorithm), were analyzed using QIIME tools (18). Alpha diversity was estimated with the Observed OTUs, Chao 1, Simpson, and Shannon index. Beta diversity was assessed by the Bray–Curtis distance and presented by principal coordinate analysis (PCA) and non-metric multidimensional scaling (NMDS). The differences between groups in taxonomic composition taxa were analyzed using the linear discriminant analysis (LDA) effect size (LEfSe) analysis, and LDA scores > 4 were defined as discriminative taxa. R package (v3.5.2) was utilized to draw diagrams.

### Statistical Analysis

Data were reported as means ± SD (*n* = 6). SPSS 16.0 was conducted to statistically analyze the obtained data. The *t*-test for unpaired results was used to evaluate differences between two groups. *P*-value < 0.05 was considered statistically significant. ^###^*p* < 0.001 vs. control, ^##^*p* < 0.01 vs. control, ^#^*p* < 0.05 vs. control; ^***^p < 0.001 vs. model, ^**^p < 0.01 vs. model, *p < 0.05 vs. model.

## Results

### Identification of Active Components of YZM

A total of fifty-five active compounds were identified through UHPLC-QE-MS, which belonged to the following chemical classes: flavonoids (14), coumarins (8), organic acids (7), phenols (6), terpenoids (4), alkaloids (3), and lactones (3), such as resveratrol, oxyresveratrol, (R)-oxypeucedanin, phloroglucinol, atractylenolide II, neocnidilide maltol, kaempferol, fisetin, isomeranzin, and allocryptopine. The identified active chemical compounds, retention time, experimental mass with positive/negative mode, formula, and class are presented in Supplementary Table 1.

### YZM Improved the Pathological States of Colitis Mice

**FIGURE 1:**
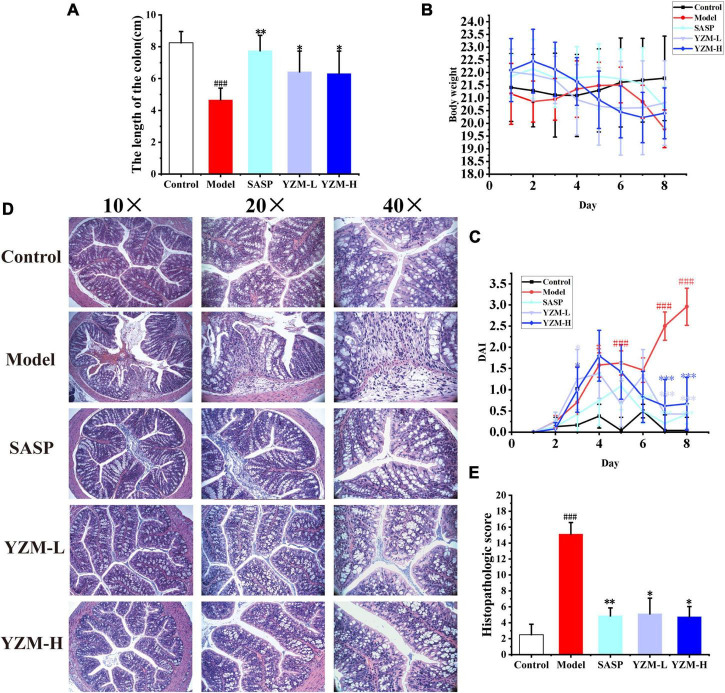
The effect of YZM on colitis mice. **(A)** Analysis of colon length. **(B)** Body weight changes. **(C)** DAI changes. **(D)** Representative images of colon sections. **(E)** Histological score. ^###^*p* < 0.001 vs. control, **p < 0.01 vs. model, *p < 0.05 vs. model.

Compared to the control group, the model group and drug intervention groups induced with DSS showed significant weight loss, shorter colon length, and decreased stool consistency (Figure 1 and Supplementary Figure 1). The colon length decreased by approximately 43.52, 6.06, 22.06, and 23.52% in the model group, SASP group, YZM-L group, and YZM-H group (Figure 1A). The weight loss of YZM-treated mice was recovered from days 5 to 8 (Figure 1B). Furthermore, the DAI scores in the SASP and YZM groups were improved (Figure 1C).

The structure of the control group was well structured with no damage to the crypt, without prominent inflammatory infiltration. The mucous membrane of the model group was widely absent, and situations of erosion, hyperemia, and edema were observed. Obvious inflammation with considerable lymphocytes infiltrated and gathered between the basal layer and mucosal muscle in the model group. There was edema in the submucosa, and a large number of goblet cells disappeared, showing classic inflammatory changes, and the damaged area of the colon accounted for more than 50% of the entire colon (Figure 1D). The histopathological score of the model group was significantly higher than other groups. Compared with the model group, mucosal inflammatory cell infiltration, erosion, and edema in YZM groups (Figure 1E) were improved significantly.

### YZM Restores the Gut Microbiota and Metabolites

#### YZM Influenced Gas Production in Fermentation

**FIGURE 2:**
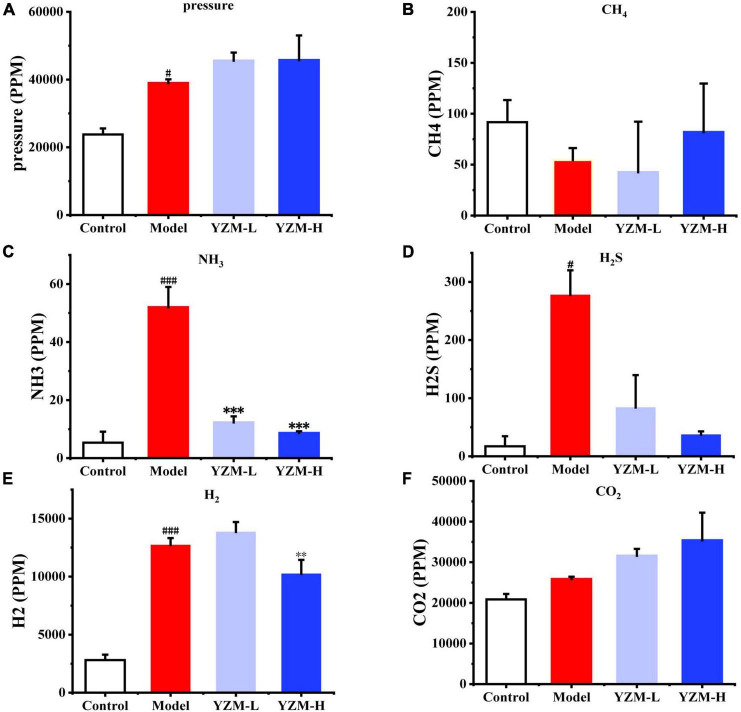
Gas produced by intestinal microbiota. **(A)** The pressure after fermentation. **(B–F)** The abundance of CH_4_, NH_3_, H_2_S, H_2_, and CO_2_, respectively. ^###^*p* < 0.001 vs control, ^#^*p* < 0.05 vs control; ***p < 0.001 vs model, **p < 0.01 vs model.

Intestinal gases produced by the microbiota could take a series of impacts on intestine homeostasis. Gas pressure and composition (CO_2_, followed by H_2_, H_2_S, CH_4_, and NH_3_) were detected as the indicators of fermentation rate. The pressure increased with the increasing YZM dose (Figure 2A), suggesting that YZM stimulated the gas production from bacteria. In Figure 2B, CH_4_ production of YZM-L was lower than in other groups but increased with the increasing concentration of YZM. The production of NH_3_ and H_2_S was inhibited in YZM-L and YZM-H groups (Figures 2C,D). In addition, H_2_ production was inhibited in the YZM-H group (Figure 2E). Since CO_2_ is the main component of intestinal gas, it shows a similar trend to gas pressure (Figure 2F).

#### YZM Restored the Production of Short-Chain Fatty Acids

**FIGURE 3:**
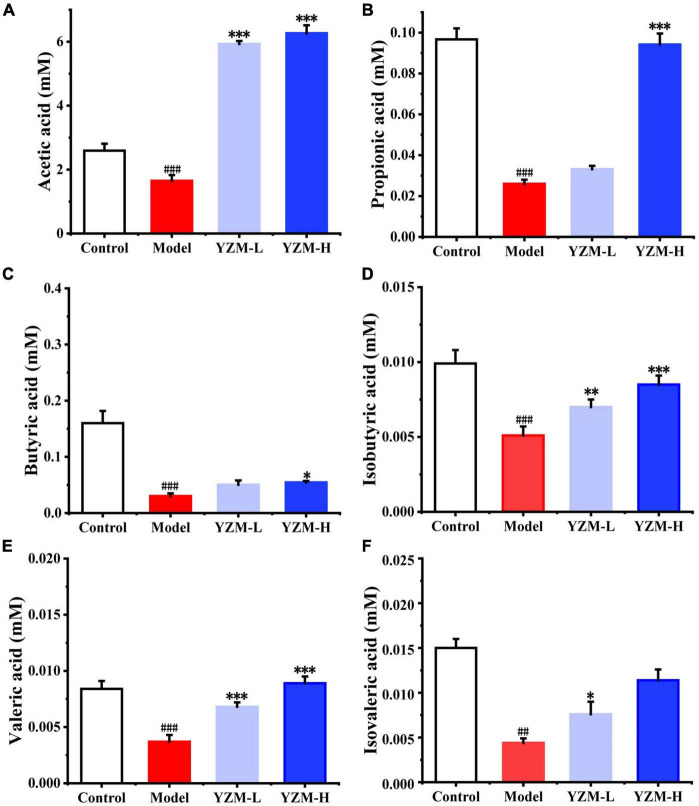
SCFAs produced by intestinal microbiota. **(A–F)** Represents SCFAs amount as acetic acid, propionic acid, butyric acid, isobutyric acid, valeric acid, Isovaleric acid. ^###^*p* < 0.001 vs control, ^#^*p* < 0.05 vs control; ***p < 0.001 vs model, **p < 0.01 vs model, *p < 0.05 vs model.

Short-chain fatty acids play crucial roles in human microorganism–host interaction and the pathogenetic mechanism of colitis. In fecal samples, we measured the SCFA (acetic, propionic, isobutyric, butyric, valeric acids, and isovaleric) level. Compared with the control group, the model group mice showed decreased contents of SCFAs (Figure 3). YZM treatment particularly increased the concentrations of acetic and propionic acids (Figures 3A,B). The contents of other measured SCFAs displayed similar increasing trends, whereas the changes were not significant in YZM-treated groups (Figures 3C–F).

#### YZM Modulated Intestinal Microbiome Composition

**FIGURE 4:**
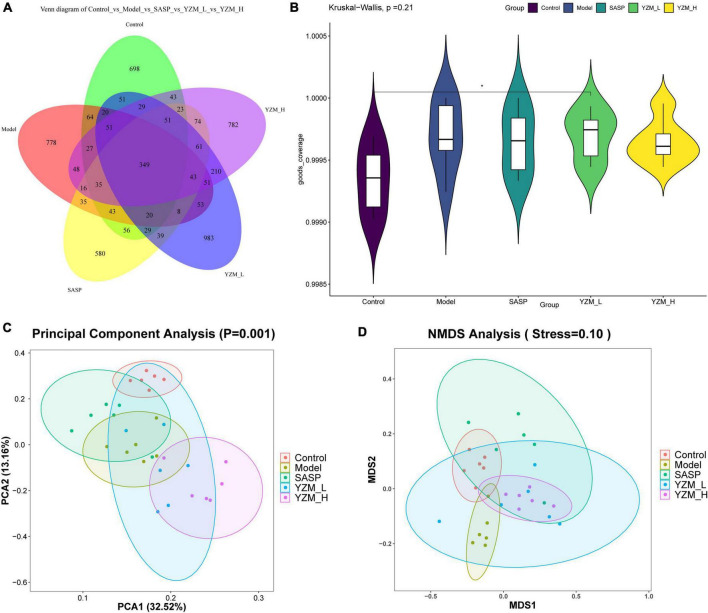
Effects of YZM on intestinal microbiota. **(A)** The cluster analysis. **(B)** The alpha diversity, **(C)** PCA. **(D)** NMDS. *p < 0.05 vs. model.

The observed species, Chao 1, Shannon index, and Simpson index of the samples were chosen to represent the alpha diversity of the microbes in the samples (Table 3) (19). These indices of YZM groups were higher than that of the model. However, these effects were not significant. These results indicated that the sum of bacterial species in the faces sample of YZM groups was higher than that in the sample of the model, to a certain extent. The microbiota alpha’s diversity in the model group was repressed, which was reversed by YZM treatment (Figures 4A,B). It was largely improved in observed OTUs and Chao1 indices after the treatment of YZM-H.

**TABLE 3:** Effects of YZM on overall structural modulation of gut microbiota.

	Control	Model	SASP	YZM_L	YZM_H
Observed OTUs	624.50 ± 103.27	505.17 ± 141.76	424.00 ± 142.15	569.83 ± 160.10	609.80 ± 73.15
Shannon	6.77 ± 0.59	6.42 ± 0.32	5.61 ± 0.28*	6.39 ± 0.76	6.59 ± 0.27
Simpson	0.97 ± 0.01	0.96 ± 0.01	0.94 ± 0.01**	0.96 ± 0.02	0.97 ± 0.01
Chao1	632.96 ± 105.22	509.10 ± 145.26	428.15 ± 145.07	572.49 ± 159.29	612.96 ± 74.63

* Indicates significant difference (**p < 0.01 vs. model, *p < 0.05 vs. model).

Principal coordinate analysis and non-metric multidimensional scaling (NMDS) showed that an apparent clustering division between the control group and model group revealed various differential OTUs (Figures 4C,D). Meanwhile, the community structures of the YZM-L group (PCA) and YZM-H (NMDS) were tended to the control group. These results implied that YZM treatment significantly promotes microbial richness.

**FIGURE 5:**
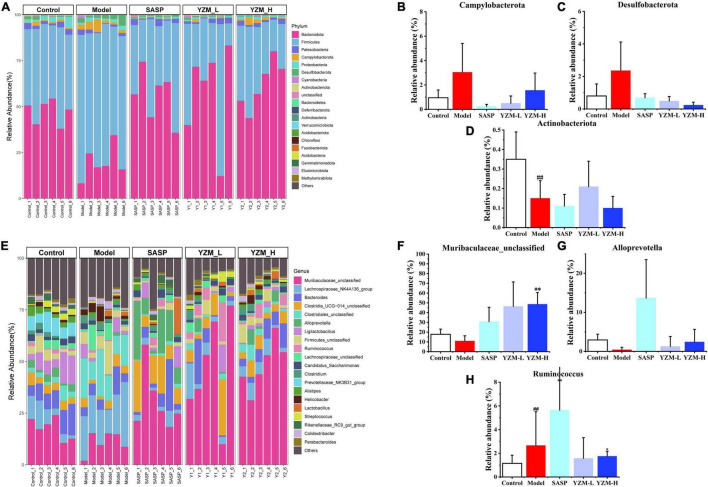
Impacts of YZM at *phylum* and *genus* level. **(A)** The proportion of intestinal microbiota at *phylum* level **(B–D)**. Relative abundance of *Campylobacterota*, *Desulfobacterota*, *Actinobacteriota*. **(E)** The proportion of intestinal microbiota at *genus* level. **(F–H)** Relative abundance of *Muribaculaceae unclassified*, *Alloprevotella*, *Ruminococcus*. ^##^*p* < 0.01 vs. control; **p < 0.01 vs. model, *p < 0.05 vs. model.

According to the differences in composition at the phylum level, *Campylobacterota* and *Desulfobacterota* displayed a higher abundance in the model group and a lower abundance of *Actinobacteriota* (Figure 5A). The relative abundance of these three taxonomic microbiotas was corrected in SASP and YZM groups (Figures 5B–D). The abundance of *Ruminococcus* increased, whereas *Muribaculaceae_unclassified* and *Alloprevotella* decreased in the model group at the genus level (Figure 5E). However, YZM-L, YZM-H, and SASP significantly reversed the intestinal bacterial composition (Figures 5F–H). This reflected that YZM administration could change the intestinal flora of colitis mice to adjust them to be a healthy biological barrier.

**FIGURE 6:**
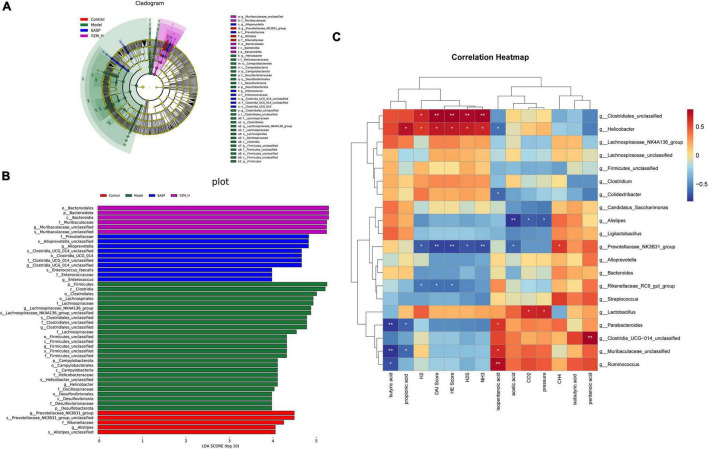
LEfSe analysis and correlation analysis. **(A)** LEfSe analysis. **(B)** LDA scores (LDA) >4, *p* < 0.05. **(C)** Heatmap of Pearson correlation analysis, Red-white-blue color: positive correlation-no correlation-negative correlation. **p < 0.01, *p < 0.05.

Consistent with other results, linear discriminant analysis effect size (LEfSe) was conducted to identify the differences in the dominant communities (Figures 6A,B). *Campylobacteria* was the key bacteria associated with intestinal microbiota disorder in the model group (LDA = 4.14, *p* = 0.003). The strain dramatically decreased once YZM and SASP intervened (Figure 5B). In the YZM-H group, *Muribaculaceae_unclassified* at genus level exhibited comparative enrichment (LDA = 5.27, *p* = 0.001). Pearson correlation analysis assessed relationships among intestinal microbiota, microbiota-derived metabolites, and gas production (Figure 6C). Potential pathogens, such as *Clostridiales*, were highly correlated with risk factors (high DAI score, HE score, H_2_S, and NH_3_), whereas beneficial bacteria, including *Prevotellaceae NK3B31*, were negatively associated with these hazard factors.

## Discussion

*Polygonatum odoratum* (Mill.) Druce (YZ) has been widely used as a food source and traditional medicine (20). It has been used to remedy various inflammatory diseases, such as flu virus, diabetes, obesity, and antitumor (21). We identified 46 compounds in YZM, flavonoids, coumarins, alkaloids, terpenoids, phenols, organic acids, and lactones, such as resveratrol. Studies show that resveratrol exerts anti-inflammatory effects within intestinal cells and prevents the onset of DSS-induced colitis (22–24). Furthermore, human clinical trials of resveratrol indicated that it improves the quality of life in patients with IBD by lowering inflammation and oxidative stress (25). Increasing evidence demonstrates that flavonoids play a critical role against IBD by modulating the gut microbiota and the metabolites (13, 26). Further studies provided an adequate theoretical basis for the anti-IBD effect of YZM. It is the first time demonstrating that *Polygonatum odoratum* could be a potential treatment for colitis. Our present results suggested that YZM, the methanol extract from *Polygonatum odoratum*, could remarkably improve colon shortening, body weight reduction, and decreased DAI score in colitis mice. H&E staining indicated that the intestinal tissue of the YZM group was similar to the control group. YZM could improve the integrity of the intestinal epithelium layer and prevent mucosal damage in DSS-induced mice. Our study lays the groundwork for developing the *Polygonatum odoratum*, a new treat from nature food and traditional Chinese medicine. In addition, an in-depth research is being developed in our laboratory.

Subsequently, we demonstrated that YZM efficiently influenced gut gas production, SCFAs synthesis, and gut microbiota composition.

The gases generated from the gut are becoming increasingly intrigued. Various gases, including H_2_, CH_4_, CO_2_, H_2_S, and NH_3_, work as the modulator of human health. In the gut, these gases are generated via the metabolic actions of resident microbiota in the colon. CO_2_ is the main product of gut microbiota and is rapidly excreted via breath, whereas it is a noble gas with volume-related mechanical stimuli (27). H_2_ is the major gas marker of carbohydrate fermentation, which is used to diagnose poor carbohydrate absorption. Studies are highlighting emerging links between H_2_ and *Ruminococcus* spp., but it is still inconclusive (28, 29). CH_4_ is generated from the metabolism of CO_2_ and H_2_ by archaea in the colon. The value of the CH_4_ profile in the breathing is more controversial than for H_2_ in clinical diagnostic tests (29). H_2_S is produced during the fermentation of proteins and has toxic impacts at high concentrations in human tissues (29). A high concentration of NH_3_ is a precipitating factor causing hepatic encephalopathy. After fermentation, the production of H_2_S and NH_3_ was inhibited considerably. The production of H_2_S and NH_3_ was significantly inhibited in YZM groups; this may be because YZM inhibited the gas-producing bacteria or modulated the microbiome composition, which inhibited the H_2_S and NH_3_ production in samples. An increase in the *Desulfobacterota* phylum has been associated with rising toxins production and bacterial genes attached to virulence agents (30). Interestingly, *Desulfobacterota* phylum’s abundance was dramatically decreased in YZM-treated compared with the model group. It is similar to H_2_S production. YZM may contribute to the decrease of H_2_S by reducing the abundance of bacteria that produce H_2_S.

*Polygonatum odoratum* on recovered the production of SCFAs in colitis mice. SCFAs are the main metabolites produced by gut microbiome fermentation and have anti-inflammatory properties and immunomodulatory effects. For the reasons mentioned above, SCFAs are critical in maintaining colon health. Studies showed that low concentrations of SCFAs were observed in colitis mice (31). In this study, the contents of measured SCFAs in model mice showed a decrease compared to control group mice. On the other hand, the high dose of YZM administration dramatically increased valeric and acetic acids. Our results suggested that YZM reversed the abundance of beneficial symbiotic and SCFA-related bacteria, such as *Muribaculaceae* (32), *Ruminococcus* (33), and *Alloprevotella* (34). The changes in SCFA-related bacteria caused by YZM might contribute to and restore SCFAs and ameliorate DSS-induced colitis.

Dextran sulfate sodium-treated mice were usually associated with the changes in the gut microbial composition as the increase in the pernicious microbe and the decrease in beneficial microorganisms (3). Our results indicated a large shift in the microbial community and changes in abundance or dominance of microbial groups under YZM treatments. *Campylobacter jejuni* disorder the protective toll-like receptor 9 (TLR9) signaling in intestinal epithelial cells and aggravated colitis in mice treated with DSS (35). *Desulfobacterota*, as a toxin bacteria, can accelerate the generation of inflammatory factors and exacerbations of colitis (30). YZM observably restored the microbiota composition by modulating phylum *Campylobacterota*, *Desulfobacterota*, and *Actinobacteriota* in DSS-treated mice. The results suggest that the *Actinobacteria* changed with YZM administration compared to the model group (36, 37). Similarly, *Actinobacteria* was raised sharply after the colitis-associated colon cancer mice were replenished with probiotics. Meanwhile, *Muribaculaceae* was related to SCFAs to tolerate immunity stimulation (38). YZM also could increase the abundance of *Muribaculaceae*. *Clostridiales* contributed to the enhanced colitis severity in chronic colitis observed in mice, and in our experiment (39), it also can increase the production of harmful gases (H_2_S and NH_3_). *Prevotellaceae* of the gut can degrade polysaccharides and high carbohydrates and benefit the disease status (40, 41). It is similar to our results, which could reverse the production of harmful gases. Altogether, we had shown that YZM evidently could alleviate colitis via reversed intestinal microbiota disorder in colitis mice.

**FIGURE 7:**
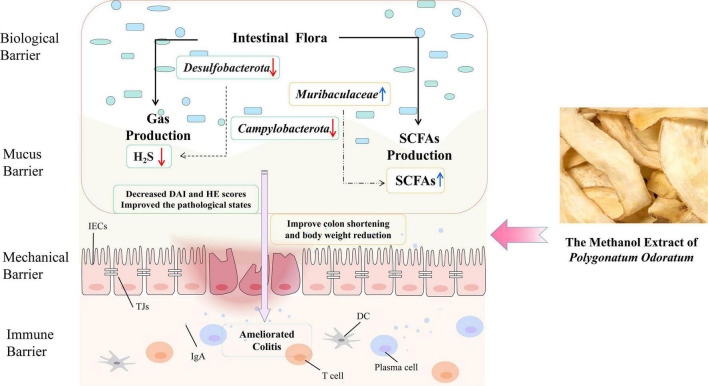
Schematic of YZM alleviating colitis.

Taken together, we found that *Polygonatum odoratum* might be a multi-targeted resource as food and medicine, for protecting against IBD (Figure 7). YZM ameliorates colonic pathological damage to relieve inflammation injure. Moreover, YZM has a modulation affection on the gut microbiome community to decrease the maleficent bacteria associated with gas production (H_2_S and NH_3_). On the other hand, it improved the intestinal microbiota’s composition and metabolites (such as SCFAs) to benefit the gut.

In summary, the methanol extract of *Polygonatum odoratum* (YZM), a plant resource used in food and medicine, was confirmed to alleviate mice colitis and is considered a novel intestinal microecological modifier with bright development prospects. The complex mechanism of YZM regulatory modulation of the intestinal immune response through microbiota needs further study.

## Data Availability Statement

The original contributions presented in the study are included in the article/Supplementary Material, further inquiries can be directed to the corresponding authors. All consensus sequence data of mice were submitted to the National Center for Biotechnology Information Short Read Archive under accession no. PRJNA817426.

## Ethics Statement

The animal study was reviewed and approved by the Ethics Committee of Zhejiang Academy of Agricultural Sciences (No. 2021ZAASLA82). Written informed consent was obtained from the owners for the participation of their animals in this study.

## Author Contributions

LL, XY, and WL contributed to the study design. XY, XP, WZ, YC, and JN conducted animal experiments. KW and LX analyzed the samples and data. All authors contributed to the article, read, and approved the submitted version.

## Conflict of Interest

The authors declare that the research was conducted in the absence of any commercial or financial relationships that could be construed as a potential conflict of interest.

## Publisher’s Note

All claims expressed in this article are solely those of the authors and do not necessarily represent those of their affiliated organizations, or those of the publisher, the editors and the reviewers. Any product that may be evaluated in this article, or claim that may be made by its manufacturer, is not guaranteed or endorsed by the publisher.

## References

[1] Zhu X Yang Y Gao W Jiang B Shi L. Capparis spinosa alleviates DSS-Induced ulcerative Colitis via regulation of the gut microbiota and oxidative stress. *Evid Based Complement Alternat Med.* (2021) 2021:1227876. 10.1155/2021/1227876 34956375 PMC8695000

[2] McGuckin MA Eri R Simms LA Florin TH Radford-Smith G. Intestinal barrier dysfunction in inflammatory bowel diseases. *Inflamm Bowel Dis.* (2009) 15:100–13. 10.1002/ibd.20539 18623167

[3] Nishida A Inoue R Inatomi O Bamba S Naito Y Andoh A. Gut microbiota in the pathogenesis of inflammatory bowel disease. *Clin J Gastroenterol.* (2018) 11:1–10. 10.1007/s12328-017-0813-5 29285689

[4] Zuo T Ng SC. The gut microbiota in the pathogenesis and therapeutics of inflammatory bowel disease. *Front Microbiol.* (2018) 9:2247. 10.3389/fmicb.2018.02247 30319571 PMC6167487

[5] Li F Han Y Cai X Gu M Sun J Qi C Dietary resveratrol attenuated colitis and modulated gut microbiota in dextran sulfate sodium-treated mice. *Food Funct.* (2020) 11:1063–73. 10.1039/c9fo01519a 31825043 PMC7122795

[6] Ji XL Guo JH Ding DQ Gao J Hao LR Guo XD Structural characterization and antioxidant activity of a novel high-molecular-weight polysaccharide from Ziziphus Jujuba cv. Muzao. *J Food Meas Charact.* [Preprint]. (2022): 10.1007/s11694-022-01288-3

[7] Ji XL Hou CY Gao YG Xue YQ Yan YZ Guo XD. Metagenomic analysis of gut microbiota modulatory effects of jujube (Ziziphus jujuba Mill.) polysaccharides in a colorectal cancer mouse model. *Food Funct.* (2020) 11:163–73. 10.1039/c9fo02171j 31830158

[8] Memariani Z Abbas SQ Ul Hassan SS Ahmadi A Chabra A. Naringin and naringenin as anticancer agents and adjuvants in cancer combination therapy: efficacy and molecular mechanisms of action, a comprehensive narrative review. *Pharmacol Res.* (2021) 171:105264. 10.1016/j.phrs.2020.105264 33166734

[9] Ji XL Hou CY Shi MM Yan YZ Liu YQ. An insight into the research concerningpanax ginsengC. A. meyer polysaccharides: a Review. *Food Rev Int.* (2020) 2020:1771363. 10.1080/87559129.2020.1771363

[10] Bauer C Duewell P Mayer C Lehr HA Fitzgerald KA Dauer M Colitis induced in mice with dextran sulfate sodium (DSS) is mediated by the NLRP3 inflammasome. *Gut.* (2010) 59:1192–9. 10.1136/gut.2009.197822 20442201

[11] Zhou D Feng Y Li W Liu B Liu X Sun L Cytotoxic steroidal glycosides from Polygonatum odoratum (Mill.) Druce. *Phytochemistry.* (2021) 191:112906. 10.1016/j.phytochem.2021.112906 34390889

[12] Pang X Zhao JY Wang YJ Zheng W Zhang J Chen XJ Steroidal glycosides, homoisoflavanones and cinnamic acid derivatives from Polygonatum odoratum and their inhibitory effects against influenza A virus. *Fitoterapia.* (2020) 146:104689. 10.1016/j.fitote.2020.104689 32726589

[13] Wu T Wang X Xiong H Deng Z Peng X Xiao L Bioactives and their metabolites from Tetrastigma hemsleyanum leaves ameliorate DSS-induced colitis via protecting the intestinal barrier, mitigating oxidative stress and regulating the gut microbiota. *Food Funct.* (2021) 12:11760–76. 10.1039/d1fo02588k 34747421

[14] Abdelhafez OH Othman EM Fahim JR Desoukey SY Pimentel-Elardo SM Nodwell JR Metabolomics analysis and biological investigation of three Malvaceae plants. *Phytochem Anal.* (2020) 31:204–14. 10.1002/pca.2883 31390115

[15] Liu Q Chen L Laserna AKC He Y Feng X Yang HS. Synergistic action of electrolyzed water and mild heat for enhanced microbial inactivation of *Escherichia coli* O157:H7 revealed by metabolomics analysis. *Food Control.* (2020) 110:107026. 10.1016/j.foodcont.2019.107026

[16] Li D Feng Y Tian M Ji J Hu X Chen F. Gut microbiota-derived inosine from dietary barley leaf supplementation attenuates colitis through PPARgamma signaling activation. *Microbiome.* (2021) 9:83. 10.1186/s40168-021-01028-7 33820558 PMC8022418

[17] Liu W Li Z Yang K Sun P Cai M. Effect of nanoemulsion loading finger citron (Citrus medica L. var. Sarcodactylis) essential oil on human gut microbiota. *J Funct Foods.* (2021) 77:104336. 10.1016/j.jff.2020.104336

[18] Logue JB Stedmon CA Kellerman AM Nielsen NJ Andersson AF Laudon H Experimental insights into the importance of aquatic bacterial community composition to the degradation of dissolved organic matter. *ISME J.* (2016) 10:533–45. 10.1038/ismej.2015.131 26296065 PMC4817675

[19] Zhao X Chen L Wongmaneepratip W He Y Zhao L Yang HS. Effect of vacuum impregnated fish gelatin and grape seed extract on moisture state, microbiota composition, and quality of chilled seabass fillets. *Food Chem.* (2021) 354:129581. 10.1016/j.foodchem.2021.129581 33756319

[20] Wang Y Fei Y Liu L Xiao Y Pang Y Kang J Polygonatum odoratum polysaccharides modulate gut microbiota and mitigate experimentally induced obesity in rats. *Int J Mol Sci.* (2018) 19:19113587. 10.3390/ijms19113587 30428630 PMC6274832

[21] Shu XS Lv JH Tao J Li GM Li HD Ma N. Antihyperglycemic effects of total flavonoids from Polygonatum odoratum in STZ and alloxan-induced diabetic rats. *J Ethnopharmacol.* (2009) 124:539–43. 10.1016/j.jep.2009.05.006 19454312

[22] Nunes S Danesi F Del Rio D Silva P. Resveratrol and inflammatory bowel disease: the evidence so far. *Nutr Res Rev.* (2018) 31:85–97. 10.1017/S095442241700021X 29191255

[23] Serra D Almeida LM Dinis TC. Anti-inflammatory protection afforded by cyanidin-3-glucoside and resveratrol in human intestinal cells via Nrf2 and PPAR-gamma: comparison with 5-aminosalicylic acid. *Chem Biol Interact.* (2016) 260:102–9. 10.1016/j.cbi.2016.11.003 27818126

[24] Wagnerova A Babickova J Liptak R Vlkova B Celec P Gardlik R. Sex Differences in the Effect of Resveratrol on DSS-Induced Colitis in Mice. *Gastroenterol Res Pract.* (2017) 2017:8051870. 10.1155/2017/8051870 28465680 PMC5390549

[25] Samsamikor M Daryani NE Asl PR Hekmatdoost A. Resveratrol supplementation and oxidative/anti-oxidative status in patients with ulcerative colitis: a randomized, double-blind, placebo-controlled pilot study. *Arch Med Res.* (2016) 47:304–9. 10.1016/j.arcmed.2016.07.003 27664491

[26] Zhang J Xu X Li N Cao L Sun Y Wang J Licoflavone B, an isoprene flavonoid derived from licorice residue, relieves dextran sodium sulfate-induced ulcerative colitis by rebuilding the gut barrier and regulating intestinal microflora. *Eur J Pharmacol.* (2022) 916:174730. 10.1016/j.ejphar.2021.174730 34968462

[27] Modak A. Breath tests with (13)C substrates. *J Breath Res.* (2009) 3:040201. 10.1088/1752-7155/3/4/040201 21386144

[28] Simmering R Taras D Schwiertz A Le Blay G Gruhl B Lawson PA Ruminococcus luti sp. nov., isolated from a human faecal sample. *Syst Appl Microbiol.* (2002) 25:189–93. 10.1078/0723-2020-00112 12353871

[29] Chatterjee S Park S Low K Kong Y Pimentel M. The degree of breath methane production in IBS correlates with the severity of constipation. *Am J Gastroenterol.* (2007) 102:837–41. 10.1111/j.1572-0241.2007.01072.x 17397408

[30] Goldstein EJ Citron DM Peraino VA Cross SA. Desulfovibrio desulfuricans bacteremia and review of human Desulfovibrio infections. *J Clin Microbiol.* (2003) 41:2752–4. 10.1128/JCM.41.6.2752-2754.2003 12791922 PMC156571

[31] Zhao Y Luan H Gao H Wu X Zhang Y Li R. Gegen Qinlian decoction maintains colonic mucosal homeostasis in acute/chronic ulcerative colitis via bidirectionally modulating dysregulated Notch signaling. *Phytomedicine.* (2020) 68:153182. 10.1016/j.phymed.2020.153182 32065953

[32] Zhao L Zhang F Ding X Wu G Lam YY Wang X Gut bacteria selectively promoted by dietary fibers alleviate type 2 diabetes. *Science.* (2018) 359:1151–6. 10.1126/science.aao5774 29590046

[33] Mottawea W Chiang CK Muhlbauer M Starr AE Butcher J Abujamel T Altered intestinal microbiota-host mitochondria crosstalk in new onset Crohn’s disease. *Nat Commun.* (2016) 7:13419. 10.1038/ncomms13419 27876802 PMC5122959

[34] Ren Y Geng Y Du Y Li W Lu ZM Xu HY Polysaccharide of Hericium erinaceus attenuates colitis in C57BL/6 mice via regulation of oxidative stress, inflammation-related signaling pathways and modulating the composition of the gut microbiota. *J Nutr Biochem.* (2018) 57:67–76. 10.1016/j.jnutbio.2018.03.005 29677563

[35] O’Hara JR Feener TD Fischer CD Buret AG. Campylobacter jejuni disrupts protective Toll-like receptor 9 signaling in colonic epithelial cells and increases the severity of dextran sulfate sodium-induced colitis in mice. *Infect Immun.* (2012) 80:1563–71. 10.1128/IAI.06066-11 22311925 PMC3318425

[36] He Y Xie Z Xu Y Zhao X Zhao L Yang H. Preservative effect of slightly acid electrolysed water ice generated by the developed sanitising unit on shrimp (Penaeus vannamei). *Food Control.* (2022) 136:108876. 10.1016/j.foodcont.2022.108876

[37] Mendes MCS Paulino DSM Brambilla SR Camargo JA Persinoti GF Carvalheira JBC. Microbiota modification by probiotic supplementation reduces colitis associated colon cancer in mice. *World J Gastroentero.* (2018) 24:1995–2008. 10.3748/wjg.v24.i18.1995 29760543 PMC5949713

[38] Biggs MB Medlock GL Moutinho TJ Lees HJ Swann JR Kolling GL Systems-level metabolism of the altered Schaedler flora, a complete gut microbiota. *ISME J.* (2017) 11:426–38. 10.1038/ismej.2016.130 27824342 PMC5270571

[39] Liu XY He SW Li QY Mu X Hu G Dong H. Comparison of the gut microbiota between pulsatilla decoction and levofloxacin hydrochloride therapy on *Escherichia coli* infection. *Front Cell Infect Mi.* (2020) 10:319. 10.3389/fcimb.2020.00319 32714880 PMC7344306

[40] Gu CH Suleria HAR Dunshea FR Howell K. Dietary lipids influence bioaccessibility of polyphenols from black carrots and affect microbial diversity under simulated gastrointestinal digestion. *Antioxidants Basel.* (2020) 9:9080762. 10.3390/antiox9080762 32824607 PMC7464840

[41] Ferrario C Statello R Carnevali L Mancabelli L Milani C Mangifesta M How to feed the mammalian gut microbiota: bacterial and metabolic modulation by dietary fibers. *Front Microbiol.* (2017) 8:1749. 10.3389/fmicb.2017.01749 28955319 PMC5600934

