# Supplementary figures and images for: The Methanol Extract of Polygonatum odoratum Ameliorates Colitis by Improving Intestinal Short-Chain Fatty Acids and Gas Production to Regulate Microbiota Dysbiosis in Mice
Source: Front Nutr. 2022 May 12;9:899421. doi: 10.3389/fnut.2022.899421 (PMC9133717; doi:10.3389/fnut.2022.899421)

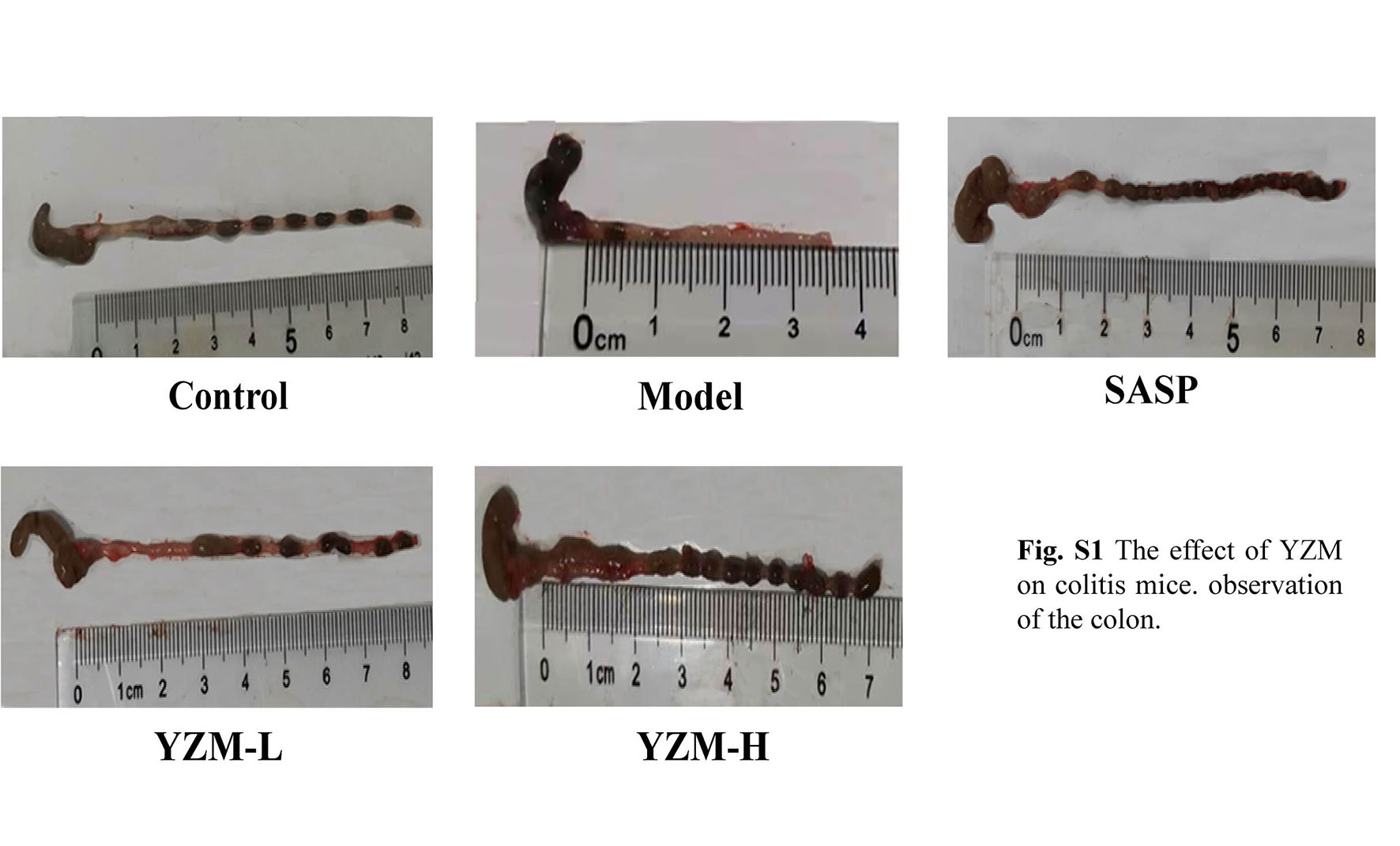

Supplement: Supplementary file 1 [file Image_1.jpg]
