# Supplementary material for: The Methanol Extract of Polygonatum odoratum Ameliorates Colitis by Improving Intestinal Short-Chain Fatty Acids and Gas Production to Regulate Microbiota Dysbiosis in Mice
Source: Front Nutr. 2022 May 12;9:899421. doi: 10.3389/fnut.2022.899421 (PMC9133717; doi:10.3389/fnut.2022.899421)
Supplement: Supplementary file 2 [file Data_Sheet_1.PDF]

Table S1. Characterization of chemical constituents of YZM by UHPLC-QE-MS

Order	RT (min)	Formula	Identify	Class	m/z	Mode	Content (%)
1	3.68	$C_{10}H_{13}N_5O_5$	Crotonoside	Alkaloids	284.1008327	Positive	2.71
2	3.79	$C_6H_6O_3$	Phloroglucinol	Phenols	127.0377097	Positive	3.95
3	6.03	$C_{15}H_{20}O_2$	Atractylenolide II	Lactones	233.1496036	Positive	1.89
4	6.84	$C_{16}H_{22}O_9$	Sweroside	Terpenoids	357.1184873	Negative	0.07
5	7.32	$C_{11}H_{22}O_2$	Undecanoic acid	Organic Acids	186.9527709	Positive	0.05
6	7.59	$C_6H_6O_3$	Maltol	Phenols	127.0388775	Positive	0.91
7	7.93	$C_{21}H_{23}NO_5$	Allocryptopine	Alkaloids	369.1619452	Positive	0.01
8	8.10	$C_9H_6O_4$	Aesculetin	Coumarins	177.0207628	Negative	0.22
9	8.33	$C_{10}H_{18}O_4$	Sebacic acid	Organic Acids	185.1169322	Positive	0.02
10	8.41	$C_{21}H_{21}O_{11}$	Cyanidin 3-glucoside	Flavonoids	449.1076966	Positive	0.01
11	8.43	$C_{11}H_{10}O_5$	Isofraxidin	Coumarins	221.0462197	Negative	0.01
12	8.52	$C_{16}H_{14}O_6$	Hesperetin	Flavonoids	301.0729802	Negative	0.02
13	8.61	$C_{14}H_{14}O_4$	Decursinol	Coumarins	247.0967706	Positive	0.01
14	8.67	$C_{15}H_{24}O$	Caryophyllene alpha-oxide	Coumarins	203.1794518	Positive	0.01
15	8.77	$C_{11}H_{13}NO_3$	N-Acetyl-L-phenylalanine	Coumarins	208.0968037	Positive	0.10
16	8.86	$C_{10}H_{10}O_4$	trans-Ferulic acid	Organic Acids	177.054075	Positive	1.93
17	8.96	$C_{28}H_{32}O_{15}$	Diosmin	Flavonoids	607.1680997	Negative	0.01
18	9.18	$C_{10}H_{16}O$	Perillyl alcohol	Terpenoids	135.1167521	Positive	0.02
19	9.36	$C_{10}H_{16}O_2$	Geranic acid	Organic Acids	169.122296	Positive	0.10
20	9.39	$C_{16}H_{12}O_5$	Glycitein	Flavonoids	283.0618173	Negative	0.01
21	9.46	$C_{14}H_{12}O_4$	Oxyresveratrol	Phenols	227.0711533	Positive	0.02
22	9.71	$C_{18}H_{16}O_8$	Rosmarinic acid	Phenols	359.0801469	Negative	0.04
23	9.82	$C_{15}H_{10}O_6$	Luteolin	Flavonoids	285.039655	Negative	0.84
24	10.16	$C_{16}H_{14}O_5$	(R)-Oxypeucedanin	Coumarins	287.0920501	Positive	0.02
25	10.22	$C_{18}H_{29}NO_3$	Dihydrocapsaicin	Alkaloids	308.2191335	Positive	1.74
26	10.4	$C_{15}H_{12}O_6$	Eriodictyol	Flavonoids	289.0704671	Positive	0.03
27	10.43	$C_{15}H_{10}O_6$	Fisetin	Flavonoids	287.0542429	Positive	0.12
28	10.44	$C_{15}H_{20}O_4$	(plusmn)Abscisic Acid	Terpenoids	247.1327571	Positive	0.10
29	10.62	$C_{15}H_{16}O_9$	Aesculin	Coumarins	341.0906359	Positive	0.19
30	10.93	$C_{18}H_{32}O_2$	Linoleic acid	Organic Acids	281.137241	Positive	0.01
31	11.24	$C_{15}H_{10}O_5$	Apigenin	Flavonoids	269.0459532	Negative	0.12
32	11.37	$C_{14}H_{12}O_3$	Resveratrol	Phenols	229.085827	Positive	0.12
33	11.38	$C_9H_6O_3$	Umbelliferone	Coumarins	161.0253146	Negative	0.04
34	11.46	$C_{16}H_{12}O_6$	Kaempferide	Flavonoids	299.0564136	Negative	1.04
35	11.56	$C_{15}H_{10}O_6$	Kaempferol	Flavonoids	285.0434633	Negative	0.59
36	11.62	$C_{12}H_{18}O_2$	Neocnidilide	Lactones	195.1379935	Positive	1.12
37	11.74	$C_{16}H_{14}O_5$	Sakuranetin	Flavonoids	285.0772975	Negative	0.20
38	12.1	$C_{10}H_{12}O$	Estragole	Phenols	149.0961802	Positive	0.02
39	12.48	$C_{21}H_{20}O_{11}$	Luteolin 7-galactoside	Flavonoids	447.1376586	Negative	0.09
40	12.57	$C_{15}H_{16}O_4$	Isomeranzin	Lactones	261.113333	Positive	0.09
41	12.71	$C_{17}H_{14}O_6$	Gnaphaliin	Flavonoids	315.0881191	Positive	0.64
42	12.8	$C_{15}H_{10}O_5$	Genistein	Flavonoids	271.1881057	Positive	0.59
43	12.87	$C_{18}H_{28}O_3$	(2E,4Z,7Z,8E)-Colnelenic acid	Organic Acids	293.2097295	Positive	2.27
44	13.5	$C_{10}H_{16}O$	Pulegone	Terpenoids	153.127426	Positive	0.33
45	13.88	$C_{22}H_{42}O_2$	Erucic acid	Organic Acids	338.3412856	Positive	77.57

RT: Retention Time
